# Adaptation of Bordetella pertussis to vaccination: a cause for its reemergence?

**DOI:** 10.3201/eid0707.017708

**Published:** 2001

**Authors:** F R Mooi, I H van Loo, A J King

**Affiliations:** National Institute for Public Health and the Environment, Bilthoven, the Netherlands. fr.mooi@rivm.nl

## Abstract

In the Netherlands, as in many other western countries, pertussis vaccines have been used extensively for more than 40 years. Therefore, it is conceivable that vaccine-induced immunity has affected the evolution of Bordetella pertussis. Consistent with this notion, pertussis has reemerged in the Netherlands, despite high vaccination coverage. Further, a notable change in the population structure of B. pertussis was observed in the Netherlands subsequent to the introduction of vaccination in the 1950s. Finally, we observed antigenic divergence between clinical isolates and vaccine strains, in particular with respect to the surface-associated proteins pertactin and pertussis toxin. Adaptation may have allowed B. pertussis to remain endemic despite widespread vaccination and may have contributed to the reemergence of pertussis in the Netherlands.


### Adaptation of *Bordetella pertussis* to Vaccination: A Cause for Its Reemergence?

**Frits R. Mooi, Inge H. M. van Loo, and Audrey J. King**
National Institute for Public Health and the Environment, Bilthoven, the Netherlands

In the Netherlands, as in many other western countries, pertussis vaccines have been used extensively for more than 40 years. Therefore, it is conceivable that vaccine-induced immunity has affected the evolution of *Bordetella pertussis*. Consistent with this notion, pertussis has reemerged in the Netherlands, despite high vaccination coverage. Further, a notable change in the population structure of *B. pertussis* was observed in the Netherlands subsequent to the introduction of vaccination in the 1950s. Finally, we observed antigenic divergence between clinical isolates and vaccine strains, in particular with respect to the surface-associated proteins pertactin and pertussis toxin. Adaptation may have allowed *B. pertussis* to remain endemic despite widespread vaccination and may have contributed to the reemergence of pertussis in the Netherlands.

*Bordetella pertussis* and *Bordetella parapertussis* are the etiologic agents of whooping cough or pertussis, a respiratory disease that is most severe in infants and young children. Compared to *B. pertussis*, *B. parapertussis* is isolated less frequently from pertussis patients (1% to 5% of pertussis patients in The Netherlands) and generally causes less severe symptoms. Pertussis is highly contagious, and, in the prevaccination era, nearly every child contracted this disease. The clinical course of pertussis is characterized by paroxysms, or bursts, of numerous, rapid coughs followed by a long inspiratory effort, which may be accompanied by a characteristic high-pitched whoop (hence the designation whooping cough). During such an attack, patients may turn blue due to lack of oxygen. In serious cases, this oxygen deprivation may lead to brain damage. The most common complication of pertussis, however, is secondary pneumonia. Young infants are at highest risk for pertussis-associated complications. More than 50% of infants less than 6 months old who contract pertussis require hospitalization. Treatment of pertussis is primarily supportive, and adequate control of the disease depends on effective immunization. Before vaccination was introduced in the 1950s, pertussis was a major cause of infant death throughout the world (1). Widespread vaccination of young children has been successful in controlling the disease (1).

### Vaccination

The high rate of illness and death caused by pertussis stimulated the early development of vaccines composed of whole, killed bacteria. These whole-cell vaccines were introduced in many countries in the 1950s and 1960s and have been highly successful in reducing the incidence of pertussis (1). The desire to avoid the side effects of whole-cell vaccines has stimulated the development of less reactogenic, acellular, vaccines composed of purified *B. pertussis* proteins (2). Acellular vaccines are replacing whole-cell vaccines in many countries.

Address for correspondence: Frits R. Mooi, Research Laboratory for Infectious Diseases (LIO), National Institute of Public Health and the Environment, P.O. Box 1, 3720 BA, Bilthoven, The Netherlands; fax: 31-30-274.4449; e-mail: fr.mooi@rivm.nl

Despite vaccination, pertussis is an endemic disease. Various sero-epidemiologic studies have shown that the frequency of infection may be as high as 1% to 4% (de Melker and Schellekens, pers. comm.) (3). Further up to 30% of people with a persistent cough were found to have been infected with *B. pertussis* (4). It is possible that vaccination initially reduced the circulation of *B. pertussis* and that adaptation allowed the *B. pertussis* population to restore its high circulation rate. This assumption predicts a change in the makeup of the *B. pertussis* population after the introduction of vaccination, a phenomenon that has indeed been observed in The Netherlands.

### Changes in *B. pertussis* in a Highly Vaccinated Population

In some countries with highly vaccinated populations such as Australia (5), Canada (6), and The Netherlands (7) (Figure 1), pertussis has reemerged. Such a phenomenon may have been caused by changes in the accuracy of notifications, decreases in vaccine coverage, or changes in vaccine quality. These possibilities have been excluded for The Netherlands (7), and we have proposed another possible cause: adaptation of *B. pertussis*

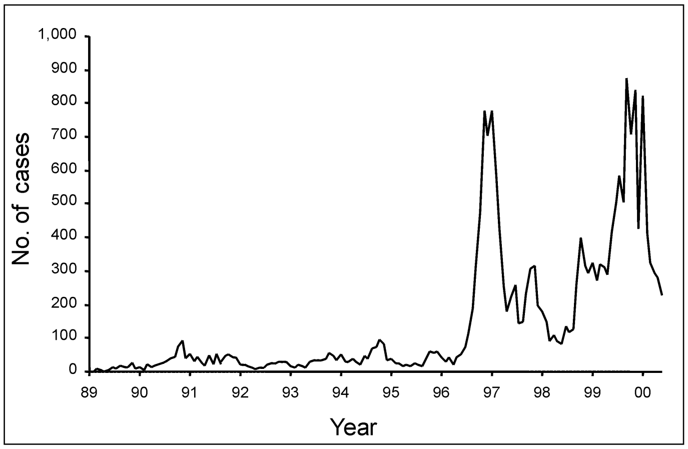

Figure 1. Notifications of pertussis in the Netherlands. Source: National Institute of Public Health and the Environment to the vaccine. To investigate this hypothesis, *B. pertussis* strains collected in The Netherlands from 1949 to 1996 were characterized by DNA fingerprinting and sequencing of genes coding for surface proteins (8,9).

Initially, we studied changes in the *B. pertussis* population by *IS1002*-based DNA fingerprinting (9,10). Strains collected from 1949 to 1996 were stratified in periods of 5 to 8 years, and the frequency of fingerprint types in each period was determined (Figure 2). Widespread vaccination was introduced in The Netherlands in 1953, and we assumed that the *B. pertussis* population was not significantly affected by vaccination from 1949 to 1954 (defined as the prevaccination period in Figure 2). Notable differences were

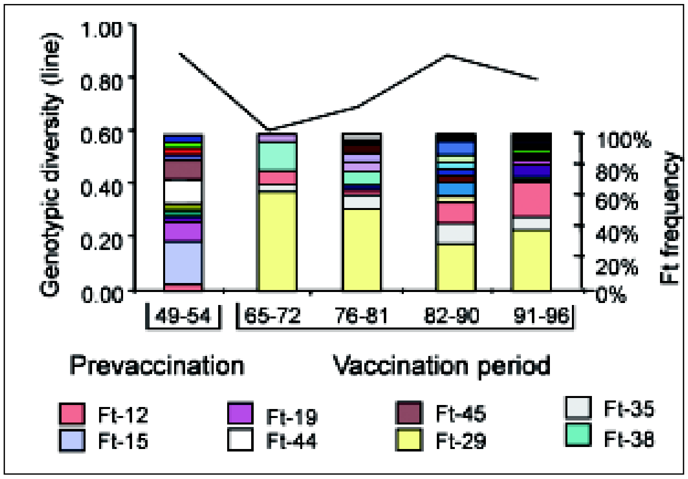

Figure 2. Changes in the population structure of *B. pertussis* in The Netherlands as determined by IS*1002*-based DNA fingerprinting. Strains isolated from Dutch patients from 1949 to 1996 were stratified in periods of 5 to 8 years. The frequency of fingerprint types (Ft) in each period was determined and is displayed by colored bars. Genotypic diversity was calculated according to Nei, using fingerprint data (11). Adapted from (10).

found between the populations from the prevaccination era and the subsequent period, both in the type and frequency of fingerprint types (e.g., the major fingerprint type found in strains collected from 1965-1972 [Ft-29] was absent during the prevaccination period). These qualitative observations were confirmed by the trend in genotypic diversity (Figure 2). Genotypic diversity decreased significantly after the introduction of vaccination and subsequently increased to prevaccination levels. In the 1980s, a second decrease in genotypic diversity occurred. Apart from sampling artifacts, a drop in genotypic diversity may be caused by a decrease in population size or clonal expansion. Indeed, we found that the reduction in genotypic diversity in the 1960s and 1980s was associated with the expansion of antigenically distinct strains.

In a second study of changes in the *B. pertussis* population, we investigated whether antigenic shifts had occurred in surface proteins (8). Very little polymorphism was observed in most proteins studied. However, two virulence factors, pertussis toxin and pertactin, were polymorphic. Interestingly, antibodies against these proteins correlate with protection against disease, which suggests they have an important role in inducing host immunity (12,13). Essentially, all DNA polymorphisms observed were nonconservative, indicating Darwinian selection. Three pertactin and three pertussis toxin variants were found in the Dutch *B. pertussis* population (Figure 3). Polymorphism in pertussis toxin was restricted to the S1 subunit (PtxS1), which carries the toxic activity. Variation in PtxS1 was observed in two regions. Significantly, one of the polymorphic residues has been implicated in binding to the T-cell receptor (14).

Polymorphism in pertactin was confined to a region comprised of tandem repeats located proximally to the RGD

```
        160
         |
Prn1    RGDAPA GGAVP GGAVP GGAVP ----- ----- GGFGP GGFGP VL
Prn2    RGDAPA GGAVP GGAVP ----- GGFGP GGFGP GGFGP GGFGP VL
Prn3    RGDAPA GGAVP GGAVP ----- ----- GGFGP GGFGP GGFGP VL

          66                227
           |                 |
PtxS1D   V L E H L   -//- R M A P V V G
PtxS1B   V L D H L   -//- R M A P V I G
PtxS1A   V L D H L   -//- R I A P V I G
```

Figure 3. Polymorphism in pertactin (Prn) and the S1 subunit of pertussis (PtxS1) toxin found in Dutch strains. The RGD sequence in pertactin, involved in binding to host receptors, has been underlined. Dashes indicate gaps. Numbers refer to positions of amino acids relative to the N-terminal methionine.

motif involved in adherence to host tissues (15). Regions with tandem repeats are known to undergo rapid variation due to slipped-strand mispairing during replication (16). Pertactin and PtxS1 variants, identical to those included in the Dutch whole-cell vaccine, were found in 100% of the strains from the 1950s, when the whole-cell vaccine was introduced in The Netherlands (Figure 4). However, nonvaccine types of pertactin and PtxS1 gradually replaced the vaccine types in later years and were found in 90% of strains collected from 1990 to 1996. These results suggest that vaccination has caused strains that are antigenically distinct from vaccine strains to be selected. The drop in genotypic diversity observed in the 1960s and the 1980s coincided with the emergence of nonvaccine-type pertussis toxin and pertactin variants, respectively, suggesting that the drop was caused by clonal expansion. Antigenic divergence between vaccine strains and clinical isolates was also observed in other

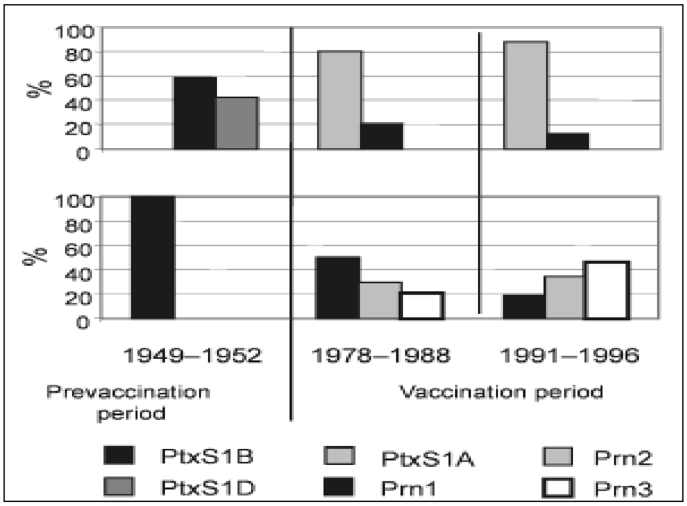

Figure 4. Temporal trends in frequencies of pertussis toxin and pertactin variants in The Netherlands.

countries with a long history of pertussis vaccination, such as Finland and the United States (17,18), and also in Italy, where vaccine coverage has varied considerably.

### Discussion

Is polymorphism in pertactin and pertussis toxin driven by host immunity, or is it the result of random fixation due to genetic drift? The latter possibility is highly unlikely since essentially all DNA mutations we detected in the pertactin and pertussis toxin genes were nonconservative. In contrast, random genetic drift is characterized by a high degree of conservative mutations in protein coding regions (20). Further, the polymorphic regions interact directly with the immune system. The polymorphic region of pertactin induces a protective immune response (unpublished data). One of the polymorphic residues in PtxS1 has been implicated in binding to the T-cell receptor (14). Finally, the fact that the same temporal trends in allele frequencies are observed in geographically distinct regions such as Finland, the United States, and The Netherlands argues against random genetic drift. It is possible that the polymorphic loci we have identified are linked to other, as yet unknown, polymorphic loci that increase fitness of strains in vaccinated populations (hitchhiking).

Strains carrying nonvaccine-type pertactin or pertussis toxin variants were not found in the prevaccination era. Although the number of strains analyzed from this period was limited, these data suggest that the nonvaccine-type variants are not able to displace the vaccine-type strains in unvaccinated populations (i.e., they have a lower fitness level, or reproductive rate, in unvaccinated communities). Alternatively, the nonvaccine-type strains may have evolved relatively recently. Consistent with the first hypothesis, we have observed that nonvaccine-type strains are less fit in naive mice than vaccine-type strains. In immune mice the difference in fitness between the two types of strains was much less pronounced (unpublished data). Thus vaccination has acted to shift the competitive balance between strains.

An important question to address is whether adaptation of the *B. pertussis* population has affected vaccine efficacy, i.e., contributed to the reemergence of *B. pertussis*. Animal experiments have indicated that variation in pertactin affects vaccine efficacy (unpublished data). Further, we found vaccine-type pertactin variants less frequently among vaccinated persons than among unvaccinated persons, which would be expected if the vaccine protects differentially against strains with distinct pertactin types (8). However, the extent to which polymorphism affects vaccine efficacy is probably dependent on the vaccine used. It is conceivable that the increase in fitness associated with nonvaccine types of pertactin and pertussis toxin in vaccinated populations is substantial enough to drive expansion of strains carrying these protein variants but that the effect is too small to result in a measurable drop in vaccine efficacy. Further studies are required to assess the effect of the observed adaptations on the efficacy of pertussis vaccines. In this period, when whole-cell vaccines are being replaced by acellular vaccines in many countries, continued strain surveillance is of paramount importance.

This research was supported by the PraeventieFonds, grant numbers 25-2545 and 28-2852, the Ministry of Health, Welfare and Culture, and RIVM.

Dr. Mooi is at the National Institute of Public Health and the Environment, The Netherlands, and the Eijkman-Winkler Laboratory of the University of Utrecht. His current interests are molecular epidemiology and evolution of *Bordetella* spp.

### References

1. Willems RJL, Mooi FR. From whole cell to acellular pertussis vaccines. Reviews in Medical Microbiology 1996;7:13-21.
2. Halparin SA. Developing better paediatric vaccines. The case of pertussis vaccine. Biodrugs 1999;12:175-91.
3. Wright SW, Decker MD, Edwards KM. Incidence of pertussis infection in healthcare workers. Infect Control Hosp Epidemiol 1999;20:120-3.
4. Cherry JD. Epidemiological, clinical, and laboratory aspects of pertussis in adults. Clin Infect Dis 1999;28:S112-7.
5. Andrews R, Herceq A, Roberts C. Pertussis notifications in Australia. Commun Dis Intell 1997;21:145-8.
6. DeSerres G, Boulianne N, Douville-Fradet M, Duval B. Pertussis in Quebec: ongoing epidemic since the late 1980s. Can Commun Dis Rep 1995;15:45-8.
7. De Melker HE, Conyn-van Spaendock MAE, Rümke HC, van Wijngaarden JK, Mooi FR, Schellekeus JFP. Pertussis in the Netherlands: an outbreak despite high levels of immunization with whole cell vaccine. Emerg Infect Dis 1997;3:175-8.
8. Mooi FR, van Oirschot H, Heuvelman K, van der Heide HGJ, Gaastra W, Willems RJL. Polymorphism in the *Bordetella pertussis* virulence factors P.69/pertactin and pertussis toxin in the Netherlands: temporal trends and evidence for vaccine-driven evolution. Infect Immun 1998;66:670-5.
9. Van der Zee A, Vernooij S, Peeters M, van Embden JDA, Mooi FR. Dynamics of the population structure of *Bordetella pertussis* as measured by IS*1002*-associated RFLP: comparison of pre- and post-vaccination strains and global distribution. Microbiology 1996;142:3479-85.
10. Van Loo IHM, van der Heide HGJ, Nagelkerke NJD, Verhoef J, Mooi FR. Temporal trends in the population structure of *Bordetella pertussis* in the years 1949-1996 in a highly vaccinated population. J Infect Dis 1999;79:915-23.
11. Nei M, Tajima F. DNA polymorphism detectable by restriction endonucleases. Genetics 1981;97:145-63.
12. Cherry JD, Gornbein J, Heininger U, Stehr K. A search for serologic correlates of immunity to *Bordetella pertussis* cough illnesses. Vaccine 1998;16:1901-6.
13. Storsaeter J, Hallander HO, Gustafsson L, Olin P. Levels of anti-pertussis antibodies related to protection after household exposure to *Bordetella pertussis*. Vaccine 1998;16:907-16.
14. De Magistris MTA, DiTommaso A, Domenighini M, Censini S, Tagliabue AJR, Oksenberg L, et al. Interaction of the pertussis toxin peptide containing residues 30-42 with DR1 and the T-cell receptors of 12 human T-cell clones. Proc Natl Acad Sci U S A 1992;89:2990-4.
15. Everest P, Li L, Douce G, Charles I, De Azavedo J, Chatfield S, et al. Role of *Bordetella pertussis* P.69/pertactin protein and the P.69/pertactin RGD motif in the adherence to and invasion of mammalian cell. Microbiology 1996;142:3261-8.
16. Streisinger G, Owen JE. Mechanisms of spontaneous and induced frameshift mutations in bacteriophage T4. Genetics 1984;109:633-59.
17. Mooi FR, Qiushui He, van Oirschot H, Mertsola J. Variation in the *Bordetella pertussis* virulence factors pertussis toxin and pertactin in vaccine strains and clinical isolates in Finland. Infect Immun 1999;67:3133-4.
18. Cassiday P, Sanden G, Heuvelman K, Mooi FR, Bisgard KM, Popovic T. Polymorphism in *Bordetella pertussis* pertactin and pertussis toxin virulence factors in the United States, 1935-1999. J Infect Dis 2000;12:1402-8.
19. Mastrantonio P, Spigaglia P, van Oirschot H, van der Heide HGL, Heuvelman K, Stefanelli P, et al. Antigenic variants in *Bordetella pertussis* strains isolated from vaccinated and unvaccinated children. Microbiology 1999;45:2069-75.
20. Ochman H, Wilson AC. Evolution in bacteria: evidence for a universal substitution rate in cellular genomes. J Mol Evol 1987;26:74-86.

## References

[PDF_00230] Andrews R., Herceg A., Roberts C. (1997). Pertussis notifications in Australia, 1991 to 1997.. Commun Dis Intell.

[PDF_00274] Cassiday P., Sanden G., Heuvelman K., Mooi F., Bisgard K. M., Popovic T. (2000). Polymorphism in Bordetella pertussis pertactin and pertussis toxin virulence factors in the United States, 1935-1999.. J Infect Dis.

[PDF_00228] Cherry J. D. (1999). Epidemiological, clinical, and laboratory aspects of pertussis in adults.. Clin Infect Dis.

[PDF_00254] Cherry J. D., Gornbein J., Heininger U., Stehr K. (1998). A search for serologic correlates of immunity to Bordetella pertussis cough illnesses.. Vaccine.

[PDF_00260] De Magistris M. T., Di Tommaso A., Domenighini M., Censini S., Tagliabue A., Oksenberg J. R., Steinman L., Judd A. K., O'Sullivan D., Rappuoli R. (1992). Interaction of the pertussis toxin peptide containing residues 30-42 with DR1 and the T-cell receptors of 12 human T-cell clones.. Proc Natl Acad Sci U S A.

[PDF_00232] De Serres G., Boulianne N., Douville Fradet M., Duval B. (1995). Pertussis in Quebec: ongoing epidemic since the late 1980s.. Can Commun Dis Rep.

[PDF_00264] Everest P., Li J., Douce G., Charles I., De Azavedo J., Chatfield S., Dougan G., Roberts M. (1996). Role of the Bordetella pertussis P.69/pertactin protein and the P.69/pertactin RGD motif in the adherence to and invasion of mammalian cells.. Microbiology.

[PDF_00278] Mastrantonio P., Spigaglia P., van Oirschot H., van der Heide H. G., Heuvelman K., Stefanelli P., Mooi F. R. (1999). Antigenic variants in Bordetella pertussis strains isolated from vaccinated and unvaccinated children.. Microbiology.

[PDF_00270] Mooi F. R., He Q., van Oirschot H., Mertsola J. (1999). Variation in the Bordetella pertussis virulence factors pertussis toxin and pertactin in vaccine strains and clinical isolates in Finland.. Infect Immun.

[PDF_00239] Mooi F. R., van Oirschot H., Heuvelman K., van der Heide H. G., Gaastra W., Willems R. J. (1998). Polymorphism in the Bordetella pertussis virulence factors P.69/pertactin and pertussis toxin in The Netherlands: temporal trends and evidence for vaccine-driven evolution.. Infect Immun.

[PDF_00252] Nei M., Tajima F. (1981). DNA polymorphism detectable by restriction endonucleases.. Genetics.

[PDF_00282] Ochman H., Wilson A. C. (1987). Evolution in bacteria: evidence for a universal substitution rate in cellular genomes.. J Mol Evol.

[PDF_00268] Streisinger G., Owen J. (1985). Mechanisms of spontaneous and induced frameshift mutation in bacteriophage T4.. Genetics.

[PDF_00225] Wright S. W., Decker M. D., Edwards K. M. (1999). Incidence of pertussis infection in healthcare workers.. Infect Control Hosp Epidemiol.

[PDF_00235] de Melker H. E., Conyn-van Spaendonck M. A., Rümke H. C., van Wijngaarden J. K., Mooi F. R., Schellekens J. F. (1997). Pertussis in The Netherlands: an outbreak despite high levels of immunization with whole-cell vaccine.. Emerg Infect Dis.

[PDF_00248] van Loo I. H., van der Heide H. G., Nagelkerke N. J., Verhoef J., Mooi F. R. (1999). Temporal trends in the population structure of Bordetella pertussis during 1949-1996 in a highly vaccinated population.. J Infect Dis.

